# A survey among Korea Medicine doctors (KMDs) in Korea on patterns of integrative Korean Medicine practice for lumbar intervertebral disc displacement: Preliminary research for clinical practice guidelines

**DOI:** 10.1186/s12906-015-0956-1

**Published:** 2015-12-07

**Authors:** Ye-sle Shin, Joon-Shik Shin, Jinho Lee, Yoon Jae Lee, Me-riong Kim, Yong-jun Ahn, Ki Byung Park, Byung-Cheul Shin, Myeong Soo Lee, Joo-Hee Kim, Jae-Heung Cho, In-Hyuk Ha

**Affiliations:** Jaseng Spine and Joint Research Institute, Jaseng Medical Foundation, 858 Eonju-ro, Gangnam-gu, Seoul Republic of Korea; Division of Clinical Medicine, School of Korean Medicine, Pusan National University, Yangsan, Republic of Korea; Medical Research Division, Korea Institute of Oriental Medicine, Daejeon, Republic of Korea; Department of Korean Rehabilitation Medicine, Kyung Hee University, Seoul, Republic of Korea

**Keywords:** Intervertebral disc displacement, Health surveys, Complementary Therapies, Integrative medicine

## Abstract

**Background:**

Patients seek Korean Medicine (KM) treatment for a broad range of complaints in Korea, but predominantly for musculoskeletal disorders. We investigated lumbar Intervertebral Disc Displacement (IDD) practice patterns of Korean Medicine doctors (KMDs) within a hospital/clinic network specializing in KM treatment of spinal disorders through survey of diagnosis and treatment methods.

**Methods:**

Questionnaires on clinical practice patterns of KM treatment for lumbar IDD were distributed to 149 KMDs on January 25th, 2015. The questionnaire included items on sociodemographic characteristics, clinical practice patterns, and preferred method of lumbar IDD diagnosis and treatment. KMDs were asked to grade each treatment method for absolute and relative importance in treatment and prognosis, and safety.

**Results:**

A total 79.19 % KMDs (*n* = 118/149) completed the survey, and results showed that integrative care mainly consisting of acupuncture, herbal medicine, Chuna manipulation, and pharmacopuncture was administered to IDD patients. The participant KMDs largely relied on radiological findings (MRI and X-ray) for diagnosis. ‘Eight principle pattern identification’, ‘Qi and Blood syndrome differentiation’ and ‘Meridian system syndrome differentiation’ theories were generally used for KM syndrome differentiation. The most frequently prescribed herbal medication was Chungpa-jun, and most commonly used Chuna technique was ‘sidelying lumbar extension displacement treatment’. IDD patients received 1.9 ± 0.3 treatment sessions/week, and KMDs estimated that an average 9.6 ± 3.5 weeks were needed for 80 % pain relief.

**Conclusions:**

This is the first study to investigate expert opinion on KM treatment of IDD. Further randomized controlled trials and clinical guidelines based on clinical practice patterns of KM are called for.

**Electronic supplementary material:**

The online version of this article (doi:10.1186/s12906-015-0956-1) contains supplementary material, which is available to authorized users.

## Background

Low back pain (LBP) is common in general and especially older populations with prevalence reported between 8-56 % in Americans [1]. In estimations of global burden of disease, LBP is the largest cause of years lost due to disability (YLD), and ranks high (6th) in disability-adjusted life years (DALYs) as well [2]. Intervertebral disc displacement (IDD) is a major cause of severe LBP. According to the 2013 National Health Insurance Statistical Yearbook, 2,555,753 Koreans sought treatment for IDD-related disorders, spending 790,097,546 won in reimbursements [3]. While a considerable number of IDD patients opt for surgery, there is a widespread cultural preference for non-invasive treatment in Korea. Patients seek Korean Medicine (KM) treatment for a broad range of complaints, but predominantly for musculoskeletal disorders in Korea [4]. Korean Medicine doctors (KMDs) are licensed to independently and mutually exclusively practice KM, and medical doctors (MDs) conventional medicine within a dual medical system in Korea. KM covers various modalities such as acupuncture, herbal medicine, cupping, Chuna manipulation and pharmacopuncture. Both MDs and KMDs are licensed by the Korean Ministry of Health and Welfare and are required to complete 2 + 4 years of undergraduate or 4 years of postgraduate courses. KM treatment for IDD mainly consists of acupuncture, herbal medicine, Chuna manipulation, and pharmacopuncture. However, with the exception of acupuncture, KM treatment incurs a wide range of substantial out-of-pocket expenses, rendering total cost and usage assessment difficult. Although the efficacy of KM treatment for LBP has been extensively studied, most are studies on single interventions and fail to reflect actual clinical practice [5–7]. As KM treatment is not highly standardized with various aspects governing its subjective diagnosis and treatment methodology, there is increasing demand in KM communities for standardized treatment protocols and clinical practice guidelines (CPGs).

CPGs are systematic guidelines devised to guide healthcare providers and patients in decision making processes [8], and discussions for expert consensus on appropriate clinical practice patterns should precede controlled trials to be incorporated in study design and CPG construction. KMDs employed within a network of hospitals/clinics specializing in KM treatment of spine disorders were surveyed to this aim. This network includes three hospitals designated by the Korean Ministry of Health and Welfare as KM hospitals specializing in spine disorders in 2014 (2nd term) and was the first and only KM hospital to be recognized to specialize in spinal disorders from 2011 to 2014 (1st term). Specialty hospitals are hospitals accredited by the Korean Ministry of Health and Welfare as advanced medical treatment providers for specific disorders or specialties (Korean Medical law Act 3, Clause 5). As of 2015, this network comprises 17 hospitals/clinics in Korea and 7 clinics in the United States, treating over 900,000 cases a year with integrative KM (acupuncture, Chuna manipulation, herbal medicine, and pharmacopuncture). Details of the integrative treatment offered at this network have been previously studied, reporting favorable outcomes [9–11]. This study is the first to our knowledge to survey KMDs with extensive knowledge and experience in spinal disorders for expert opinion and clinical practice pattern investigation.

## Methods

### Initial draft

Six KMD researchers, all of whom completed 2 + 4 year undergraduate courses, participated in questionnaire construction (including 4 KM rehabilitation specialists with average 10+ years of clinical experience (JSS, JHL, IHH, MRK) and 2 residents (YSS, YJA) majoring in KM rehabilitation with 3+ years clinical experience). To reduce possibility of selection bias, a systematic search was conducted using PubMed. The keywords ‘LBP/herniated disc AND survey/questionnaire/clinical decision/consensus’ were used, and criteria for inclusion were articles: (1) published within the last 10 years, (2) reporting questionnaires distributed to medical/healthcare professionals and providers, and (3) written in English. Titles and abstracts were compiled for initial screening, and full texts were retrieved and read, yielding 55 relevant articles. Of the 55 articles, 19 provided partial or full questionnaire contents [12–29], all of which are referred to as model questionnaires in our study. The remaining 36 papers that did not provide questionnaire contents were further reviewed for additional information appropriate for inclusion (YSS). Suitable items were selected for consideration, and the primary researchers (IHH, MRK, YSS, YJA) debated on the optimal format for each item. For items on non-KM treatment methods, the content was revised to meet KM practice standards. Any selection/modification was finalized when 3 or more persons of the 4 researchers concurred. The first draft was completed as a 40-item questionnaire with alternating free response and multiple choice sections based on the model questionnaire items.

After initial compilation and drafts, the questionnaire was revised by each researcher individually (YSS- > YJA- > MRK- > IHH). Each researcher left notes regarding content and item format on a Microsoft Office Word file (Microsoft®, Redmond, WA, USA) in assigned colors, and passed the file on to the next researcher. After individual review, the researchers convened to share opinions on the rationale and reasoning behind each revision, and revisions were retained if agreed on by 3 or more out of the 4 researchers in collective decision. The drafted questionnaire was further reviewed by 2 KMDs (JSS, JHL).

The questionnaire was designed to guarantee sufficient anonymity and convenient and accurate data collection. As free responses leave room for ambiguity and missing values and multiple choices may restrict diversity, both forms were employed. Multiple choice answer choices were extracted from KM rehabilitation textbooks used by all 11 colleges of KM and 1 specialized graduate school in Korea, and a recent CPG on KM treatment of IDD [30–32]. Upon review of the literature, items considered to conflict with actual clinical practice by 3 or more of the 4 researchers were revised or deleted. Also, items not reported in the literature but considered necessary by the majority of researchers were added.

Items regarding acupuncture/pharmacopuncture treatment were formatted to conform to Standards for Reporting Interventions in Clinical Trials of Acupuncture (STRICTA) guidelines. While Chuna manipulation of non-spinal/pelvic areas may also have positive therapeutic effects on IDD, evidence was insufficient to establish a firm association. Therefore only Chuna manipulation techniques applied directly to the lumbar spine, ilium, sacrum, pubis, or coccyx regions were included as answer choices.

A 5-point Likert scale was initially considered for measurement of ranges (e.g. level of importance, short/long term treatment effect, and safety), but the scope was considered to be too narrow and a 7-point scale was therefore used. However, if all items shared same or similar levels of significance, it would be difficult to discern relative importance. Item ranking was included for this reason and in items where relative importance was considered to be of more relevance, ranking was used to measure ordinal importance. Hypothesizing that treatment methods possess different short and long term effects, opinions on short and long term effects were surveyed separately.

Statements extracted from two questionnaires conducted in MDs [13] were translated, revised, and added as the last section of the questionnaire to investigate level of consensus on IDD among KMDs.

The first draft was reviewed by a statistician (KBP) for items potentially unsuitable for statistical analysis. Although item ranking is not an accepted form of statistical data collection, the format was kept for multidimensional data collection and future reference.

### Second draft

The initial draft was sent electronically to a panel of 5 extramural experts. The panel consisted of a KM rehabilitation professor at a KM university, a KM rehabilitation professor at a specialized KM graduate school/chairman of a spine manipulation society, a researcher at the Korean Institute of Oriental Medicine (KIOM, a government funded research center for KM and subsidiary organization of the Korea Research Council of Fundamental Science and Technology under the Korean Ministry of Science, Information & Communication Technology and Future Planning), a researcher at KIOM/acupuncture specialist, and a methodologist, all of whom are licensed KMDs. Comments and suggested changes from panel members were compiled, and the 4 primary researchers convened for discussion. The final questionnaire was completed after 5 additional meetings, and the final version was printed after statistician approval.

### Distribution and collection of questionnaires

The questionnaires were distributed at a monthly conference for KMDs practicing at KM hospitals/clinics specializing in spinal disorders held on January 25th, 2015. A 60 min session was allotted for survey completion. KMDs in internship/residency programs also participated but were excluded from analysis as the majority did not have IDD outpatient treatment experience. Questionnaires for absentees who had notified the organizing committee of their absence in advance were prepared and delivered by way of coworkers. The completed questionnaires were asked to be returned by mail.

The study protocol received approval from the Institutional Review Boards of Jaseng Hospital of Korean Medicine in Korea (KNJSIRB2015-03), and all investigators adhered to the Helsinki Declaration. Written informed consent was obtained by informing participants of study objectives, construction process, instructions for filling out the survey, confidentiality of personal information, and use of results for academic means. Signatures were not obtained to maintain anonymity, and responding to the survey was considered written informed consent. The full survey is available in Word file format [see Additional file 1].

### Data entry

A statistician (KBP) created a Microsoft Office Excel version 14.0 (Microsoft®, Redmond, WA, USA) data sheet and instructed 2 researchers with no affiliation to this study (JHL2, WKK) on specifics of data entry. JHL2 and WKK entered the data accordingly, and on completion KBP conducted a full inspection and marked any illegible or ambiguous responses for reinvestigation (YSS). Questions not allowing for multiple responses with two or more or no numbers checked were regarded to be missing data.

### Statistical analysis

Using descriptive statistics, continuous variables were presented as mean ± SD and categorical data as frequency (%). Likert scales were analyzed as continuous variables. Plural responses were allowed for in most categorical data, and ranking scores were collected and analyzed separately. SPSS PASW statistics software version 18.0 (IBM Corporation, NY, USA) was used for all statistical analyses.

## Results

The response rate was 79.19 % (*n* = 118/149, of which 96 were collected on site, and 22 by mail). However, one respondent appeared to have misunderstood the entire questionnaire, and was excluded from analysis. The final analysis included 117 questionnaires.

KMDs participating in the study were 38.6 ± 6.2 year old males with 12.1 ± 5.5 years’ clinical experience, and of these, 31 respondents had practiced for 15+ years. Inquiry on highest level of education revealed that 24.5 % had acquired bachelor’s, 35.8 % master’s, and 39.8 % Ph. D. degrees. A total 76.4 % were specialists certified by the Korean Ministry of Health and Welfare, and (a) the Society of Korean Medicine Rehabilitation in 31.9 %; (b) the Korean Acupuncture and Moxibustion Medicine Society in 28.7 %; and (c) the Society of Internal Korean Medicine in 25.5 %. Nearly all participants (97.6 %) replied that they had received training outside of formal mandatory education, and 91.1 % of respondents had completed courses offered by the Korean Society of Chuna Manual Medicine for Spine and Nerves.

The surveyees treated 16.1 ± 7.2 IDD patients/day, and patients visited the outpatient department 1.9 ± 0.3 times/week. Respondents replied that they anticipated 4.3 ± 1.9 weeks of treatment to be needed for 50 % pain relief, and 9.6 ± 3.5 weeks for 80 %. Average duration of integrative treatment sessions administered by KMDs (encompassing Chuna manipulation, cupping, acupuncture, pharmacopuncture, and consultation) per patient was about 17.8 ± 10.9 min/session. In commonly used intervention type, acupuncture was used 100 %, Chuna 97.6 %, herbal medicine 95.9 %, and pharmacopuncture 95.9 %. Almost all outpatients received integrative care including acupuncture, Chuna, herbal medicine, and pharmacopuncture (Table 1).

**Table 1:** Demographic characteristics and clinical practice patterns of Korean Medicine doctors surveyed

Factors		mean ± sd/n (%)
Age (years)		38.6 ± 6.2
	30-39	85 (69.7)
	40-49	27 (22.1)
	≥50	10 (8.2)
Gender		
	Male	123 (100)
	Female	0 (0)
Clinical experience (years)		12.1 ± 5.5
	5 ≤ ≤10	44
	11 ≤ ≤15	48
	16 ≤ ≤20	19
	≥21	12
Level of healthcare facility of currently affiliated institution^a^		
	Primary clinic	39 (32)
	Secondary facility	83 (68)
Highest academic degree		
	Bachelor’s	30 (24.4)
	Master’s	44 (35.8)
	Ph. D.	49 (39.8)
Specialist training		
	Yes (specialist)	94 (76.4)
	No	29 (23.6)
Specialty (if applicable)		
	The Society of Korean Medicine Rehabilitation	30 (31.9)
	Korean Acupuncture and Moxibustion Medicine Society	27 (28.7)
	The Society of Internal Korean Medicine	24 (25.5)
	Other	13 (13.8)
Extracurricular training^b^		
	Yes	120 (97.6)
	No	3 (2.4)
Number of lumbar intervertebral disc displacement outpatients/day		16.1 ± 7.2
Usage rate of treatment (multiple responses allowed)		
	Acupuncture	123 (100)
	Chuna manipulation	120 (97.6)
	Herbal medicine	118 (95.9)
	Pharmacopuncture	118 (95.9)
	Cupping	109 (88.6)
	Bee venom pharmacopuncture	98 (79.7)
	Moxibustion	2 (1.6)
Average length of treatment needed for 50 % pain decrease (weeks)		4.3 ± 1.9
Average length of treatment needed for 80 % pain decrease (weeks)		9.6 ± 3.5

Reference: Evidence Based Korean Medicine Clinical Practice Guideline Development Commitee for Lumbar Herniated Intervertebral Disc (Korea Institute of Oriental Medicine, The Society of Korean Rehabilitation): Korean Medicine Clinical Practice Guideline for Lumbar Herniated Intervertebral Disc in adults (KMCGP_Lumbar Herniated Intervertebral Disc). Daejeon, Korea, 2014

^a^Primary clinics hold <30 beds for inpatient care

Secondary facilities hold 30 ≤ and <500 beds for inpatient care, and at least 4 outpatient departments including specialties

^b^Curriculum refers to 6 years of education provided at KM universities or 4 years of post-graduate courses provided at a specialized KM graduate school, a prerequisite for all certified KMDs

The most influential factors on prognosis, as determined on 7-point Likert scales, were ‘clinical symptoms’ (6.4 ± 0.9), ‘radiological findings’ (5.9 ± 1.1), and ‘time elapsed since onset and cause of onset’ (5.8 ± 1.2). Of individual treatment modalities prescribed and administered by KMDs, bee venom pharmacopuncture (BV) was regarded to be most influential in the short term (8 weeks), followed by acupuncture/pharmacopuncture and herbal medicine. In the long term (1 year), herbal medicine was considered most influential, followed by Chuna, acupuncture, and pharmacopuncture (Table 2).

**Table 2:** Influence of factors in prognosis determination and importance of individual treatment methods of lumbar intervertebral disc displacement

Prognostic factors	Importance	Treatment methods	Short term (8 weeks) importance	Long term (1 year) importance
	mean ± sd		mean ± sd	mean ± sd
Clinical symptoms^a^	6.4 ± 0.9	Bee venom pharmacopuncture	6.2 ± 1	5.4 ± 1.3
Radiological findings	5.9 ± 1.1	Acupuncture	6.1 ± 1	5.6 ± 1.3
Time elapsed since onset and cause of onset	5.8 ± 1.2	Pharmacopuncture	6.1 ± 0.9	5.6 ± 1.2
Patient attitude toward and perception of disorder	5.6 ± 1.1	Herbal medicine	6 ± 1	6.5 ± 0.8
Past history (e.g. surgery, trauma)	5.6 ± 1.1	Chuna manipulation	5.7 ± 1.1	5.6 ± 1.2
Age	5.2 ± 1.2	Cupping	4.6 ± 1.4	4 ± 1.4
Personality and other psychological factors (e.g. depression, anxiety)	5.2 ± 1.1	Moxibustion	3.9 ± 1.5	4.1 ± 1.6
Physical examination	5.1 ± 1.4			
Comorbidities	4.2 ± 1.3			
Korean Medicine syndrome differentiation	4.0 ± 1.6			

^a^Factor most frequently ranked 1st

(Importance: 1 = not important at all, 2 = unimportant, 3 = somewhat unimportant, 4 = neither important nor unimportant, 5 = somewhat important, 6 = important, 7 = very important)

KMDs referred to test results in 92.7 ± 14.1 % of patients on initial visit, and 70.0 ± 28.2 % of return visits. MRIs (98.4 %) were most frequently referred to in diagnostic testing (including diagnostic imaging and lab tests), followed by X-rays (95.1 %) and CTs (61 %), showing that imaging was the predominantly used diagnostic tool. Of lab results, C-reactive protein (CRP) was most frequently referred to (10.6 %), followed by erythrocyte sedimentation rate (ESR) (5.7 %). When reading MRI images, KMDs tended to look for ‘degree of nerve compression’ (89.4 %), ‘degree of intervertebral disc displacement’ (84.6 %), and ‘correlations between levels of disc displacement on MRI and clinical symptoms’ (71.5 %). In physical examinations relevant to lumbar IDD, straight leg raise (SLR) was most frequently performed (96.7 %), with manual muscle testing (52.8 %), and heel/toe walk (32.5 %) following. The most frequently considered KM syndrome differentiation theory was ‘Eight Principle Pattern identification (八綱辨證)’ at 70.7 %, followed by ‘Qi and Blood syndrome differentiation (氣血辨證)’ (69.9 %) and ‘Meridian system syndrome differentiation (經絡辨證)’ (68.3 %). In correlations with IDD and ‘10 Types of LBP’ from ‘Dongeuibogam’, more than half associated symptomatic LBP due to IDD with ‘LBP from Blood stagnation (瘀血腰痛)’ (56.1 %), followed by ‘LBP from Phlegm (痰飮腰痛)’ (36.6 %), and ‘LBP from contusion (挫閃腰痛)’ (33.3 %) (Table 3).

**Table 3:** Diagnostic tools most frequently used for lumbar intervertebral disc displacement and Korean Medicine syndrome differentiation of symptoms

Factors		n (%)
Tests	Magnetic resonance imaging (MRI)^a^	121 (98.4)
	X-ray	117 (95.1)
	Computed tomography (CT)	75 (61)
	C-reactive protein (CRP)	13 (10.6)
	Electromyogram	11 (8.9)
	Digital infrared thermal imaging (DITI)	9 (7.3)
	Erythrocyte sedimentation rate (ESR)	7 (5.7)
Main points of consideration when reading MRI images	Degree of nerve compression	110 (89.4)
	Degree of intervertebral disc displacement^a^	104 (84.6)
	Correlations between levels of disc displacement on MRI and clinical symptoms	88 (71.5)
	Number and level of displaced discs (e.g. L1/2 vs. L5/S1)	23 (18.7)
	Degree of intervertebral disc degeneration	14 (11.4)
	Alignment of vertebrae	12 (9.8)
	Degree of degeneration of vertebral body and/or joints (spondylosis)	9 (7.3)
	Diameter/area of spinal canal	9 (7.3)
Physical examination	Straight leg raise test (SLR)^a^	119 (96.7)
	Manual muscle testing (MMT)	65 (52.8)
	Heel walk/toe walk	40 (32.5)
	Valsalva test	29 (23.6)
	Well leg raise test	28 (22.8)
	Sensory testing	28 (22.8)
	Bragard test	22 (17.9)
	Laseque sign	13 (10.6)
	Other	23 (18.6)
Korean Medicine syndrome differentiation theories	Eight principle pattern identification (八綱辨證)^a^	87 (70.7)
	Qi and Blood diagnosis (氣血辨證)	86 (69.9)
	Meridian system diagnosis (經絡辨證)	84 (68.3)
	Organ system diagnosis (臟腑辨證)	53 (43.1)
	Six meridian diagnosis (六經辯證)	24 (19.5)
	Sasang constitutional medicine diagnosis (四象體質辨證)	21 (17.1)
	Defensive Qi and nutrient Blood diagnosis (衛氣營血辨證)	7 (5.7)
10 Types of LBP from ‘Dongeuibogam’	LBP from Blood stagnation (瘀血腰痛)^a^	69 (56.1)
	LBP from Phlegm (痰飮腰痛)	45 (36.6)
	LBP from contusion (挫閃腰痛)	41 (33.3)
	LBP from Kidney deficiency (腎虛腰痛)	25 (20.3)
	LBP from Wind pathogen (風腰痛)	19 (15.4)
	LBP from Dampness pathogen (濕腰痛)	13 (10.6)
	LBP from Dampness-Heat pathogen (濕熱腰通)	13 (10.6)
	LBP from Cold pathogen (寒腰痛)	9 (7.3)
	LBP from Qi(氣腰痛)	9 (7.3)
	LBP from retention of food (食積腰痛)	5 (4.1)

^a^Factor most frequently ranked 1st

The most frequently prescribed herbal medicine for IDD was Chungpa-jun (99.2 %), followed by Hwalhyeoljitong-tang (46.3 %) and Ojeok-san (33.3 %). ‘Sidelying lumbar extension displacement treatment technique’ (39 %), ‘sidelying lumbar ‘pitch and roll’ distraction method’ (35.8 %), and ‘prone posteriorly rotated ilium/sidebent sacrum treatment technique’ (34.1 %) were the most frequently applied Chuna manipulation techniques. Ah-shi points (91.9 %) and Motion Style Acupuncture Therapy (MSAT) (91.1 %) were the most frequently used styles and points of acupuncture, and 74 % reported symptomatic use of acupuncture for symptom relief. Regarding pharmacopuncture type, Shinbaro 1 and 2 pharmacopuncture were most frequently used (69.9 %), followed by Shinbaro 3 pharmacopuncture (44.6 %). The most commonly used acupoints were Hyeopcheok (Huatuo Jiaji, EXB2) in 66.7 %, GB30 in 52.0.%, and Ah-shi points in 43.9 % for acupuncture, and Hyeopcheok in 74.0 %, Ah-shi points in 35.8 %, and BL23 in 26.0 % for pharmacopuncture (Table 4).

**Table 4:** Frequently prescribed Korean Medicine treatments for lumbar intervertebral disc displacement

Factors		n (%)
Herbal medicine	Chungpa-jun^a^	122 (99.2)
	Hwalhyeoljitong-tang(活血止痛湯)	57 (46.3)
	Ojeok-san (五積散)	41 (33.3)
	Dokhwalgisaeng-tang (獨活寄生湯)	37 (30.1)
	Danggwisoo-san (當歸鬚散)	28 (22.8)
	Jakyagkamcho-tang (芍藥甘草湯)	24 (19.5)
	Yookmijihwang-tang (六味地黃湯)	24 (19.5)
Chuna manipulation	Sidelying lumbar extension displacement correction technique	48 (39)
	Sidelying lumbar ‘pitch and roll’ distraction method	44 (35.8)
	Prone posteriorly rotated ilium/sidebent sacrum correction technique	42 (34.1)
	Prone leg raise ilium correction technique	38 (30.9)
	Prone lumbosacral joint distraction method	34 (27.6)
	Prone sacrum sidebent rotation displacement correction technique	30 (24.4)
	Spine flexion distraction method: Flexion shift technique	25 (20.3)
	Sidelying lumbar flexion displacement correction technique	23 (18.7)
	Spine flexion distraction method: Extension technique	12 (9.8)
Style of acupuncture	Ah-shi points	113 (91.9)
	Motion Style Acupuncture Treatment (MSAT)^a^	112 (91.1)
	Acupoints relevant to symptoms (acupoints related to specific disorder/syndromes)	91 (74)
	Dong-Si Acupuncture	18 (14.6)
Pharmacopuncture	Shinbaro 1	86 (69.9)
	Shinbaro 2^a^	86 (69.9)
	Shinbaro 3	55 (44.7)
	Hwangryunhaedok	36 (29.3)
	Joongseongouhyul	30 (24.4)
	Muscle relaxation	12 (9.8)
	Anti-inflammation	11 (8.9)
	Scolopendra	11 (8.9)
Acupoints used for acupuncture	Hyeopcheok (Huatuo Jiaji, EXB2) points	82 (66.7)
	GB30 (環跳)	64 (52.0)
	Ah-shi points	54 (43.9)
	BL23 (腎兪)	44 (35.8)
	BL25 (大腸兪)	25 (18.7)
	BL40 (委中)	23 (18.7)
Acupoints used for pharmacopuncture	Hyeopcheok (Huatuo Jiaji, EXB2) points	91 (74.0)
	Ah-shi points	44 (35.8)
	BL23 (腎兪)	32 (26.0)
	GB30 (環跳)	27 (22.0)
	BL25 (大腸兪)	25 (20.3)

^a^Factor most frequently ranked 1st

The main target of acupuncture treatment was the anatomical structure most likely to cause symptoms (77.2 %), tender points, trigger points, and other points that elicit a painful response upon palpation (54.5 %), and spinal levels of pathology as confirmed through imaging (50.4 %). Acupuncture was administered with 11 ± 3.7 needles per session, inserted to a depth of 3.0 ± 1.3 cm using needles 0.3 ± 0.03 mm thick. Needle retention time was 13.9 ± 2.1 min. De qi sensation and muscle twitch responses were both considered important (rated 5.5 ± 1.4 and 5.2 ± 1.4, respectively). Manual needle stimulation such as MSAT, ‘lifting and thrusting (提揷)’, and ‘holding and twisting (捻轉)’ were widely used. Electroacupuncture was performed in 91.1 ± 20.2 % of patients. Most KMDs regarded ‘physical stimulation of solution’ to be the most influential aspect of pharmacopuncture treatment, followed by ‘chemical efficacy of solution’. An average 1.2 ± 0.8 ~ 3.2 ± 1.9 cc of pharmacopuncture solution was injected at 2.9 ± 2.2 to 5.8 ± 3.1 acupoints using 1.6 ± 1.0 to 3.6 ± 1.3 cm length needles, which took 2.2 ± 2.9 to 4.3 ± 4.2 min per patient per session to administer (Table 5).

**Table 5:** Acupuncture and pharmacopuncture treatment frequently used for lumbar intervertebral disc displacement: data collected and reported according to STRICTA standards

STRICTA checklist items		Acupuncture			Pharmacopuncture	
Acupuncture rationale	1a) Style of acupuncture	Refer to Table 4.		1a) Type of pharmacopuncture	Refer to Table 4.	
	1b) Reasoning for treatment provided	Anatomical structure likely to cause symptoms (e.g. shortened quadratus lumborum, shortened psoas muscles)^a^	95 (77.2)	1b) Reasoning for treatment provided	Physical stimulation of solution (i.e. irrigation of inflamed area, desensitization effect triggered by pain elicited by injection)	123 (100)
		Tender points, trigger points, and other points that elicit a painful response upon palpation	67 (54.5)			
		Spinal levels of pathology as confirmed through imaging (e.g. site of disc herniation)	62 (50.4)		Chemical efficacy of solution (i.e. pharmaceutical effect from major ingredients)^a^	120 (97.6)
		Ah-shi points (site of pain)	44 (35.8)			
		Effective acupoints as observed through clinical experience	43 (35)		Acupuncture effects of pharmacopuncture needle (i.e. effect from pharmacopuncture needle itself)	115 (93.5)
		Acupoints based on Korean Medicine principles (e.g. GB30, BL40, BL57)	30 (24.4)			
		Academic knowledge derived from research articles, clinical practice guidelines	15 (12.2)		Placebo effect (i.e. effect from patient anticipation)	7 (5.7)
		Knowledge acquired through formal education	13 (10.6)			
Details of needling	2a) Number of needle insertions per subject per session		11 ± 3.7	2a) Number of acupoint injections per subject per session (range)		2.9 ~ 5.8
				2a) Amount of pharmacopuncture solution injected per session (range, cc)		1.2 ~ 3.2
	2b) Names of points used	Refer to Table 4.		2b) Names of points used	Refer to Table 4.	
	2c) Depth of insertion (cm)		3.0 ± 1.3	2c) Depth of insertion (range, cm)		1.6 ~ 3.6
	2d) Responses sought	De qi sensation	5.5 ± 1.4			
		Muscle twitch response	5.2 ± 1.4			
	2e) Needle stimulation	Motion Style Acupuncture Treatment (MSAT)	69 (56.1)			
		Lifting and thrusting (提揷)	60 (48.8)			
		Holding and twisting (捻轉)	58 (47.2)			
		Percentage of patients treated with electroacupuncture (%)	91.1 ± 20.2			
	2f) Needle retention time (minutes)		13.9 ± 2.1			
	2 g) Needle type	Diameter of needle (mm)	0.3 ± 0.03			
Treatment Regimen	3a) Number of treatment sessions	Refer to Table 1.		3a) Number of treatment sessions	Refer to Table 1.	
	3b) Frequency of treatment sessions (sessions/week)		1.9 ± 0.3	3b) Frequency of treatment sessions (sessions/week)		1.9 ± 0.3
	3b) Duration of treatment sessions (minutes)		17.8 ± 10.9	3b) Duration of treatment sessions (minutes)	2.2 ~ 4.3 2.2 ± 2.9	4.3 ± 4.2
Other components of treatment	4a) Other interventions administered	Refer to Table 1.		4a) Other interventions administered	Refer to Table 1.	
Practitioner background	5) Description of participating acupuncturists	Refer to Table 1.		5) Description of participating acupuncturists	Refer to Table 1.	

^a^Factor most frequently ranked 1st

Regarding prognosis, 82 % of KMDs responded that engaging in everyday activities is likely to aggravate symptoms in most people, and that LBP/leg pain due to IDD can be alleviated without surgery (93.5 %). Approximately 46.3 % responded that back pain and leg pain is likely to improve after surgery if given sufficient time. The statement that bed rest can help some people recover from pain was supported by 95.1 % of respondents, and over-the-counter medication was considered to be effective by 81.3 %. In comparisons between non-invasive and surgical treatment, 55.7 % of KMDs regarded non-invasive treatment to be more time-efficient, and 14.8 % viewed the 2 forms to be equally effective. Almost all KMDs shared the opinion that without surgery, 3.3 ± 9.1 out of 100 IDD patients would experience permanent loss of motor function in the lower extremities, and that in 5 years, non-invasive treatment would be more effective than surgery (99.2 %) (Table 6).

**Table 6:** Knowledge items and physician opinions on clinical decisions

Statements		mean ± sd/n (%)
1. For most patients with lumbar intervertebral disc displacement, how likely is doing normal activities to make their herniated disc symptoms worse?	Likely	100 (82)
	Not very likely	22 (18)
2. Without surgery, over time, do back and leg pain caused by lumbar intervertebral disc displacement usually improve, stay the same, or deteriorate?	Improves	115 (93.5)
	Stays the same	6 (4.9)
	Deteriorates	2 (1.6)
3. With surgery, over time, do back and leg pain caused by lumbar intervertebral disc displacement usually improve, stay the same, or deteriorate?	Improves	57 (46.3)
	Stays the same	36 (29.3)
	Deteriorates	30 (24.4)
4. Can lots of bed rest help relieve pain in some patients with pain caused by lumbar intervertebral disc displacement?	Yes	116 (95.1)
	No	6 (4.9)
5. Can over-the-counter pain medicine help relieve pain in some patients with pain caused by lumbar intervertebral disc displacement?	Yes	100 (81.3)
	No	23 (18.7)
6. Which treatment is more likely to provide swifter relief from pain caused by lumbar intervertebral disc displacement?	Non-invasive care	68 (55.7)
	Surgery	36 (29.5)
	Both are similar	18 (14.8)
7. Of 100 patients who receive surgery for lumbar intervertebral disc displacement, about how many patients will experience equal or more back or leg pain after surgery?	(number of people)	23.3 ± 17.7
8. Of 100 patients who receive surgery for lumbar intervertebral disc displacement, about how many patients will experience serious complications within 3 months of surgery?	(number of people)	12.5 ± 13.7
9. Without surgery, about how many patients with lumbar intervertebral disc displacement will develop permanent loss of motor function severe enough to keep them from walking?	(number of people)	3.3 ± 9.1
10. In the long term (5 years), which treatment is better at relieving pain caused by lumbar intervertebral disc displacement?	Non-invasive care	121 (99.2)
	Surgery	-
	Both are similar	1 (0.8)

KMDs graded safety levels of treatment on a 7-point scale as follows: acupuncture 6.5 ± 0.8, pharmacopuncture 6.0 ± 0.8, BV 4.4 ± 1.1, Chuna 5.6 ± 1.0, herbal medicine 6.1 ± 0.9, cupping 6.2 ± 1.0, and moxibustion 5.1 ± 1.3 (1 = very unsafe, 2 = unsafe, 3 = somewhat unsafe, 4 = neither safe nor unsafe, 5 = somewhat safe, 6 = safe, 7 = very safe). The KMDs were in general agreement that the safest form of treatment was acupuncture, and that treatment requiring most precaution was BV. Potential adverse events included pneumothorax after acupuncture, bleeding and vascular injury after pharmacopuncture, allergic reactions including pruritus and rashes after BV administration, aggravation of pain after Chuna, gastrointestinal disorders after herbal medicine intake, and blisters and burns after cupping and moxibustion.

## Discussion

Acupuncture is included in recommendations for chronic LBP from the National Institute for Health and Care Excellence (NICE) and the American College of Physicians (ACP) and American Pain Society (APS) guidelines, which could be taken to reflect widespread acupuncture use for LBP [33, 34]. Various randomized controlled trials (RCTs) have studied the effects of acupuncture for treatment of IDD, most of which are single-intervention acupuncture or pharmacopuncture studies. However, IDD treatment in Korea is not usually singular [6, 11, 35], resulting in disparity between research and clinical settings.

This study is a current report on actual integrative KM practice patterns of IDD. Expert opinion on factors important for prognosis, frequent methods of diagnosis, and most effective treatment for IDD was surveyed in a KM network specializing in spinal disorders. We hope that such information on clinical practice patterns may act as signposts in shaping the future course of research and CPGs. There are no previous studies, to the best of our knowledge, on KMD perspective of diagnosis, treatment, and outcome evaluation of IDD. This is also the first investigation focusing on clinical practice pattern evaluation of integrative KM care for IDD patients. The participant KMDs responded that they treated 16.1 ± 7.2 IDD patients/day, and that 4.3 ± 1.9 and 9.6 ± 3.5 weeks of treatment were required for 50 % and 80 % pain reduction, respectively. Although comparison with previous reports is inappropriate as the present investigation was a survey, a prior study on conservative treatment reported average duration of pain to be 4+ months [36].

An integrative treatment regimen mainly consisting of acupuncture, herbal medicine, Chuna, and pharmacopuncture was applied to patients. KMDs reported that they most frequently referred to imaging test results, especially MRIs and X-rays. CRP was the most frequently referred lab test, and clinical symptoms and radiological findings were most regularly consulted for prognosis, which partly explains the high referral rate to radiological testing. CRP was considered to be an important marker for differential diagnosis such as infection. Ah-shi and MSAT were the dominant styles of acupuncture used, and Shinbaro 1 and 2 the preferred types of pharmacopuncture. In physical examination, SLR was regularly checked, and traditional KM syndrome differentiation relied largely on the ‘Eight Principle Pattern identification (八綱辨證)’, ‘Qi and Blood syndrome differentiation (氣血辨證)’, and ‘Meridian system syndrome differentiation (經絡辨證)’ theories. In the traditional ‘10 Types of LBP’ from ‘Dongeuibogam’, ‘LBP from Blood stagnation (瘀血腰痛)’ was regarded to most closely resemble LBP symptoms from IDD. The most commonly prescribed herbal medication was Chungpa-jun, and most frequently used Chuna technique was the ‘sidelying lumbar extension displacement treatment’. In determining prognosis of IDD patients, KMDs were shown to base decisions on ‘clinical symptoms’, ‘radiological findings’, and ‘time elapsed since onset and cause of onset’.

These results suggest that the KMDs who participated in our survey considered clinical symptoms and radiological findings to be more influential in lumbar IDD prognosis than syndrome differentiation. In additional analysis of difference in herbal medicine use by major syndrome differentiation (data not shown), results showed that Chungpa-jun was most frequently prescribed regardless of syndrome differentiation. The anti-inflammatory [37], nerve regeneration [38], and cartilage protective effects [39] of Chungpa-jun have been demonstrated in in vivo and in vitro studies, and outcomes of clinical trials using Chungpa-jun in IDD [40] and arthritis patients [41] have also been published. It can be inferred that the participant KMDs viewed evidence-based herbal medicine use to be of more relevance than syndrome differentiation.

### Strengths

The most significant strength of this study is that it is the first thorough and extensive investigation of clinical practice patterns and KMD opinion of integrative KM care of IDD. Although diagnostic imaging is required for IDD diagnosis, Korean medical law confines use of such appliances and command of medical technicians to MDs, restricting practice rights of non-integrative KM facilities. Therefore, this study surveyed KMDs practicing at integrative institutions equipped with diagnostic appliances and specializing in treatment of spinal disorders. All KMDs treating outpatients in a KM network for spine conditions were included as potential surveyees. Specialty hospitals are designated every 3 years under Act 3, Clause 5 of Korean medical law: “Designation and Evaluation of Specialty Hospitals”. There are currently 111 KM and conventional medicine specialty hospitals in Korea. Our observations revealed that respondents saw many IDD patients a day, probably owing to the fact that they practiced in a network of hospitals/clinics specializing in spine disorders. This heightens credibility of the results, especially with regard to prognosis determination and effective clinical practice patterns.

Answer choices were collected from university textbooks and other academic resources for objectivity, and clinicians and researchers with extensive experience in spinal disorders drafted and revised the questionnaire with statistical guidance.

In addition, the response rate is high compared to other surveys. Other studies collected questionnaires via e-mail or mail, whereas this study asked participants to complete the questionnaire on site during an educational conference. Response rates for additional questionnaires collected by mail were also high, which may in part owe to shared understanding of the study value.

### Limitations

However, this study is not without limitations. Although responses of participant KMDs reflect expert opinion, the study is not fully inclusive of the KMD population or opinion. All institutions within this network of hospitals/clinics operate under a common treatment protocol, and all KMD practitioners receive mandatory training consisting of clinical case presentations and hands-on sessions conducted twice a week at individual hospitals/clinics and collective monthly seminars. The surveyed KMDs were professionals trained in a standardized manner, and displayed consistent preferences for specific herbal medicine and pharmacopuncture prescriptions. Future studies surveying larger KMD populations from diverse backgrounds will further contribute to descriptive analysis of KM practice in Korea.

Another limitation is the fact that data relied on memory and subjective opinion, thus liable to bias, and should be interpreted with caution. For example, in determining average length of treatment needed for 50 % and 80 % pain reduction in clinical practice, there are many contributing factors to consider (e.g. age, comorbidities, chronicity, severity of disc herniation and symptoms). However, for those unfamiliar to KM treatment of IDD and researchers interested in efficacy of KM treatment for IDD, these results can provide a basic idea as to what treatments are used and what duration to expect clinically perceivable change. Use of prescription data entered into an electronic database is recommended for future studies for more objective practice pattern and preference evaluation.

The questionnaire used in this study also falls short in several aspects. Most items were presented as 7-point scales to provide sufficient range. However, responses were generally grouped around 4, 5, and 6, suggesting positive bias. Also, answer choices were grouped together by category, suggesting that respondents may have marked the scales in relative comparison with whatever modality they considered to be most effective, resulting in relative as opposed to absolute scores. Additionally, the extensive length of the questionnaire may have been cause for missing data. This was more evident in free response questions, especially those on treatments with reportedly low usage rates. This may be due to negative attitude toward certain treatments or a desire to avoid discussing unfamiliar topics. For example, moxibustion was an intervention of low usage for which missing data was highly frequent. Researchers should contemplate methods that minimize strain and inconvenience of participants in future survey studies. Other minor limitations include small spacing in the printed version, giving cause for unintentional plural response, and that scale ratings were interpreted as continuous variables [42].

Items from previous studies were referred to in clinical decision making, but they were not validated versions. Also, the original articles were surveys targeted at a mostly surgical population, while this survey was designed specifically for KMDs. Statements were translated into Korean and accordingly modified to reflect general KM clinical practice patterns, but not validated. Still, considerable disparities regarding opinions on surgery can be observed. The majority of both surgeons (88 %) and KMDs (93.5 %) responded that most patients can improve without surgery, but 75 % of surgeons advocated surgery in treatment option more likely to provide swift pain relief, opposed to 29.5 % of KMDs. Regarding post-surgical back and leg pain, 94 % of surgeons responded that improvement will occur in due time, whereas only 46.3 % of KMDs were of the same opinion. Decisive conclusions or comparisons cannot be drawn as previous studies report different perspectives at different timepoints, and interpretation of these results should be limited to confirmation of difference in opinion of clinical perception. The conservative treatments described in most conventional treatment studies is distinctly different from the KM-based treatment described in this study, thus weakening grounds for direct comparison. Another interesting point of difference is that while surgeons responded that engaging in daily activities is not likely to aggravate IDD in most patients (69 %), KMDs perceived everyday activity as a possible pain exacerbating factor (82 %). Also, bed rest was supported by 81.3 % of KMDs, while 75 % of surgeons did not recognize its value in accordance with current guidelines. Though evidence discourages bed rest for IDD, the respondents of this survey recommended patients to refrain from activities that may aggravate pain symptoms until conditions were stabilized through non-invasive care. Another possibility is that the wording may have been altered during translation. Further investigations on KMD perception of bed rest and daily activities using validated statements are required [43].

### Future implications

Survey studies attending to these limitations and larger KMD populations are warranted. A CPG on KM treatment of IDD has recently been published, but paucity of research limits the strength of evidence and recommendations of treatment methods [30]. Despite the high availability of reviews and clinical guidelines, how many practitioners manage patients in everyday care has been shown to be disparate from guidelines in the US, Canada, Australia, Spain, and Israel [44–48] Moreover, various methods of clinical guideline knowledge transfer to physicians and patients were found to be ineffective in improving guideline concordance [44]. Therefore, characteristics of the diagnostic process and management outlined in this study should be given more consideration when designing clinical trials and constructing clinical guidelines on IDD to facilitate implementation.

## Conclusions

This is the first study to investigate clinical practice patterns of integrative KM treatment for IDD including diagnosis, prognosis, expected duration of treatment, and approach toward integrative care with details on acupuncture and pharmacopuncture treatment. Further consideration should be given to clinical practice patterns of other causes of LBP and musculoskeletal disorders and common empirical treatment methods to the aim of constructing CPGs supported by a stronger and wider evidence base.

## Additional file

Additional file 1:
**Clinical practice of Korean medicine for lumbar intervertebral disc displacement: A survey.** The final questionnaire used for collection of data. (DOCX 87 kb)

## Abbreviations

LBP: Low back pain
YLD: Years lost due to disability
DALY: Disability-adjusted life year
IDD: Intervertebral disc displacement
KM: Korean Medicine
KMD: Korean Medicine doctor
MD: Medical doctor
CPG: Clinical practice guideline
STRICTA: Standards for Reporting Interventions in Clinical Trials of Acupuncture
KIOM: Korean Institute of Oriental Medicine
BV: Bee venom pharmacopuncture
CRP: C-reactive protein
ESR: Erythrocyte sedimentation rate
SLR: Straight leg raise
MSAT: Motion Style Acupuncture Therapy
NICE: National Institute for Health and Care Excellence
ACP: American College of Physicians
APS: American Pain Society
RCT: Randomized controlled trial
