# Supplementary material for: A survey among Korea Medicine doctors (KMDs) in Korea on patterns of integrative Korean Medicine practice for lumbar intervertebral disc displacement: Preliminary research for clinical practice guidelines
Source: BMC Complement Altern Med. 2015 Dec 7;15:432. doi: 10.1186/s12906-015-0956-1 (PMC4672541; doi:10.1186/s12906-015-0956-1)
Supplement: Additional file 1: — Clinical practice of Korean medicine for lumbar intervertebral disc displacement: A survey. The final questionnaire used for collection of data. (DOCX 87 kb) [file 12906_2015_956_MOESM1_ESM.docx]

# Clinical practice of Korean medicine for lumbar intervertebral disc displacement

# : A survey

**[Objectives]**

**By collecting data and opinions on clinical practice patterns of Korean Medicine treatment of lumbar intervertebral disc displacement, we hope to:**

1. **Establish evidence for standardization of Korean Medicine treatment**
2. **Establish the basis for future clinical practice guidelines on lumbar intervertebral disc displacement**
3. **Investigate differences between guideline and actual clinical practice**

Clinical practice guidelines (CPGs) are guidelines constructed from evidence presented in the form of randomized controlled trials (RCTs) and systematic reviews (SRs). However, RCTs and SRs have yet to cover all disorders and relevant treatment methods, and guidelines synthesized from trials results cannot fully reflect clinical practice. Furthermore, RCTs and SRs are generally labor intensive and time-consuming requiring much time and resources. Systematic collection of clinician opinion has been proposed as an alternative method of constructing guidelines. However, there is insufficient information on clinician opinion in Korean Medicine treatment of spinal disorders, and we hope that data collected through this survey may form the foundation as basic material for Korean Medicine CPGs on spinal disorders.

**[Questionnaire construction process]**

This survey was designed and constructed with reference to 55 published articles on surveys conducted in medical/healthcare providers of lumbar intervertebral disc displacement treatment. Original questionnaires were compiled for a first draft, and the questionnaire was completed through discussions and revisions based on individual modifications by 4 Korean Medicine doctors employed at a Korean Medicine hospital specializing in spinal disorders and comments from 5 extramural experts. Sample answers provided are based on various references including 『Oriental Rehabilitation Medicine 3^rd^ edition (The Society of Korean Medicine Rehabilitation)』, 『Chuna Medicine 2^nd^ edition (Korean Society of Chuna Manual Medicine for Spine & Nerves)』, and 『Lumbar Herniated Intervertebral Disc in Adults: Korean Medicine Clinical Practice Guideline (Korea Institute of Oriental Medicine)』, and additional answer choices were limited to items considered necessary after sufficient discussion.

**[Instructions]**

While the majority of questions are multiple-choice, some require you to rank items or give short responses. Please feel free to add comments should you feel there is a more appropriate answer.

We appreciate the time and effort taken to participate in this survey. Your answers will contribute to a foundation for standardization of Korean Medicine care for spinal disorders. The contents of this questionnaire will be used in statistical analysis for academic purposes only, and any personal information will remain strictly confidential other than for statistical analyses.

**Jaseng Spine and Joint Research Center, Jaseng Medical Group**

| **No.** |  |
| --- | --- |

**[PART 1. Demographic information]**

**1. Age:** ______ years old

**2. Gender:** ① Male ② Female

**3. Years of clinical experience (including years of residency, and service as public health doctor or medical officer):** ______ years

**4. Under what category is your affiliated institution classified?**

① Primary healthcare institution (clinic/private hospital under 30 inpatient beds)

② Secondary healthcare institution (middle-scale hospital/conforming to general hospital standards with 30 to 500 inpatient beds)

**5. Where is your affiliated institution located?**

| ① Seoul ② Busan ③ Incheon ④ Daegu ⑤ Gwangju ⑥ Daejeon  ⑦ Ulsan ⑧ Gyunggi ⑨ Gangwon ⑩ Chungbuk ⑪ Chungnam ⑫ Jeonbuk ⑬ Jeonnam  ⑭ Gyeongbuk ⑮ Gyeongnam ⑯ Jeju |
| --- |

**6. Do you have any experience practicing at a specialty hospital for spinal disorders as designated by the Korean Ministry of Health and Welfare?**

① Currently practicing ② Have practiced in the past ③ Have never practiced

**7. Highest academic degree:** ① Bachelor’s degree ② Master’s degree ③ Ph. D.

**8. Specialist training:** ① Yes (specialist) ② No (general practitioner) ③ Currently in residency (resident)

**8-1.** **Specialty** (Check if applicable)

| ① Korean Medicine Rehabilitation ② Korean Acupuncture and Moxibustion Medicine  ③ Oriental Neuropsychiatry ④ Internal Korean Medicine  ⑤ Korean Medicine Obstetrics and Gynecology ⑥ Korean Medicine Ophthalmology  ⑦ Sasang Constitutional Medicine ⑧ Korean Oriental Pediatrics |
| --- |

**9. Extracurricular Korean Medicine or conventional medicine training (received at academic societies, or through certification programs)**

| ① Korean Academy of Sports Oriental Medicine ② The Korea Association of Herbology  ③ Association of Spinal Manipulation & Diagnostic Method  ④ The Society of Stroke on Korean Medicine  ⑤ The Society of Korean Medicine for Obesity Research ⑥ The Society of Hyungsang Medicine  ⑦ Korea Immuno-Yakchim Society  ⑧ Korean Society of Chuna Manual Medicine for Spine & Nerves  ⑨ Korean Medicine Association of Clinical Sanghan-Geumgwe  ⑩ Korea Pharmacopuncture Institute ⑪ Other: ______________________________ **[multiple responses allowed]** |
| --- |

※Top 10 Korean Medicine societies in number of members listed in the Society of Korean Medicine as of January 2015

**[PART 2. Clinical practice patterns]**

**1. The following questions concern clinical practice patterns in outpatient care. Please fill in the following blanks to reflect your practice.**

| **Lumbar intervertebral disc displacement** |
| --- |
| A. Average number of outpatients: ______ patients/day  B. Average number of treatment sessions: ( ) times/week  C. Type of intervention: a. Acupuncture b. Pharmacopuncture c. Bee venom pharmacopuncture  d. Herbal medicine e. Chuna f. Moxibustion g. Cupping **[multiple responses allowed]**  D. Average duration of treatment per visit: ( ) minutes  E. Average length of treatment needed for 50% pain decrease: ( ) weeks  F. Average length of treatment needed for 80% pain decrease: ( ) weeks |

**[PART 3. Tests and Prognostic factors]**

**1. The following factors are known to influence diagnosis and prognosis of lumbar intervertebral disc displacement patients. Please rate the importance of individual factors on patient prognosis, and in the blank space provided below, rank the factors from most influential. (Mark as A~K)**

**(Importance: 1=not important at all, 2=unimportant, 3=somewhat unimportant,**

**4=nether important nor unimportant, 5=somewhat important, 6=important, 7=very important)**

| **Lumbar intervertebral disc displacement** | |
| --- | --- |
| **Factors** | **Importance** |
| A. Age | 1 2 3 4 5 6 7 |
| B. Past history (e.g. surgery, trauma) | 1 2 3 4 5 6 7 |
| C. Time elapsed since onset and cause of onset | 1 2 3 4 5 6 7 |
| D. Comorbidities | 1 2 3 4 5 6 7 |
| E. Radiological findings | 1 2 3 4 5 6 7 |
| F. Clinical symptoms | 1 2 3 4 5 6 7 |
| G. Korean Medicine syndrome differentiation | 1 2 3 4 5 6 7 |
| H. Physical examination | 1 2 3 4 5 6 7 |
| I. Personality and other psychological factors (e.g. depression, anxiety) | 1 2 3 4 5 6 7 |
| J. Patient attitude toward and perception of disorder | 1 2 3 4 5 6 7 |
| K. Other: _____________________________ | 1 2 3 4 5 6 7 |
| **Rank: 1st ( ) 2nd ( ) 3rd ( )** | |

**2. The following tests are relevant to lumbar intervertebral disc displacement patients. Please rank tests in the order of those you most commonly refer to. (Mark as A~Q)**

| **Lumbar intervertebral disc displacement** |
| --- |
| A. X-ray  B. Myelography  C. Discography  D. Computed Tomography (CT)  E. RI test  F. Magnetic Resonance Imaging (MRI)  G. Fluoroscopy  H. Sonography  I. Electromyogram  J. Digital Infrared Thermal Imaging (DITI)  K. C-Reactive protein (CRP)  L. Alkaline Phosphatase (ALP)  M. Creatinine  N. Creatine kinase (CK)  O. Rheumatoid Factor (RF)  P. Erythrocyte Sedimentation Rate (ESR)  Q. Other: ______________________________ |
| **1st ( ) 2nd ( ) 3rd ( )** |

※Reference: 『Oriental Rehabilitation Medicine, 3rd edition (The Society of Korean Medicine Rehabilitation)』 p27, p29

**3. The following questions regard how much you make use of the referred test results. Considering a patient you check for any test results to be a patient “checked”, please fill in the following blanks.**

3-1. About what percent of new patients do you check for test results? ____________%

3-2. For about what percent of returning patients do you check for test results? ____________%

**4. Please rank the following points of consideration in the order you consider most important when reading MRIs of a lumbar intervertebral disc displacement patient. (Mark as A~I)**

| **Lumbar intervertebral disc displacement** |
| --- |
| A. Degree of intervertebral disc displacement  B. Degree of nerve compression  C. Diameter/area of spinal canal  D. Number and level of displaced discs (e.g. L1/2 vs. L5/S1)  E. Degree of intervertebral disc degeneration  F. Degree of degeneration of vertebral body and/or joints (spondylosis)  G. Correlations between levels of disc displacement on MRI and clinical symptoms  H. Alignment of vertebrae  I. Schmorl’s nodule |
| **1st ( ) 2nd ( ) 3rd ( )** |

**5. The following physical examinations are relevant to patients with lumbar intervertebral disc displacement. Rank the tests from most commonly applied to reflect your practice. (Mark as A~U)**

| **Lumbar intervertebral disc displacement** |
| --- |
| A. Straight leg raise test (SLR)  B. Well leg raise test  C. Laseque sign  D. Crossed Laseque sign  E. Flip test  F. Bragard test  G. Femoral stretch test)  H. Kemp’s test  I. Milgram’s test  J. Valsalva test  K. Dejerene’s triad  L. Brudzinski test  M. Superficial cremasteric reflex  N. Superficial anal reflex  O. Deep tendon reflex  P. Babinski reflex  Q. Heel walk/toe walk  R. Manual muscle testing  S. Sensory testing  T. Romberg test  U. Other: ______________________________ |
| **1st ( ) 2nd ( ) 3rd ( )** |

※Reference: 『Oriental Rehabilitation Medicine, 3rd edition (The Society of Korean Medicine Rehabilitation)』, 『An evidence-based clinical guideline for the diagnosis and treatment of lumbar disc herniation with radiculopathy (North American Spine Society)』

**[PART 4. Korean Medicine syndrome differentiation]**

**1. The following theories are used for Korean Medicine syndrome differentiation. Rank the following theories in the order of most relevant when diagnosing a lumbar intervertebral disc displacement patient. (Mark as A~H)**

| **Lumbar intervertebral disc displacement** |
| --- |
| A. Eight principle pattern identification (八綱辨證)  B. Qi and Blood syndrome differentiation (氣血辨證)  C. Organ system syndrome differentiation (臟腑辨證)  D. Meridian system syndrome differentiation (經絡辨證)  E. Defensive Qi and nutrient Blood syndrome differentiation (衛氣營血辨證)  F. Six meridian syndrome differentiation (六經辯證)  G. Sasang constitutional medicine syndrome differentiation (四象體質辨證)  H. Other: _____________________________ |
| **1st ( ) 2nd ( ) 3rd ( )** |

※Reference: 『Oriental Rehabilitation Medicine, 3rd edition (The Society of Korean Medicine Rehabilitation)』 p19

**2. Of the following 10 Types of LBP (十種腰痛), select the type(s) you consider best correlates to lumbar intervertebral disc displacement symptoms.**

| **Lumbar intervertebral disc displacement** |
| --- |
| ① LBP from Kidney deficiency (腎虛腰痛)  ② LBP from Phlegm (痰飮腰痛)  ③ LBP from retention of food (食積腰痛)  ④ LBP from contusion (挫閃腰痛)  ⑤ LBP from Blood stagnation (瘀血腰痛)  ⑥ LBP from Wind pathogen (風腰痛)  ⑦ LBP from Cold pathogen (寒腰痛)  ⑧ LBP from Dampness pathogen (濕腰痛)  ⑨ LBP from Dampness-Heat pathogen (濕熱腰通)  ⑩ LBP from Qi (氣腰痛)  ⑪ Other: __________________________ |

※Reference: 『Dongeuibogam』 Ten Types of LBP

**[PART 5. Korean Medicine treatment]**

**1. Grade the following Korean Medicine treatment methods used for lumbar intervertebral disc displacement by how effective each type of treatment is in the short term (8 weeks) / long term (1 year)**.

**(Therapeutic effects: 1=very ineffective, 2=ineffective, 3=somewhat ineffective,**

**4=neither effective nor ineffective, 5=somewhat effective, 6=effective, 7=very effective)**

| **Lumbar intervertebral disc displacement** | | |
| --- | --- | --- |
| **Type of intervention** | **Treatment effects** | |
|  | **Short term (8 weeks)** | **Long term (1 year)** |
| **Herbal medicine** | 1 2 3 4 5 6 7 | 1 2 3 4 5 6 7 |
| **Chuna** | 1 2 3 4 5 6 7 | 1 2 3 4 5 6 7 |
| **Bee venom** | 1 2 3 4 5 6 7 | 1 2 3 4 5 6 7 |
| **Pharmacopuncture** | 1 2 3 4 5 6 7 | 1 2 3 4 5 6 7 |
| **Acupuncture** | 1 2 3 4 5 6 7 | 1 2 3 4 5 6 7 |
| **Moxibustion** | 1 2 3 4 5 6 7 | 1 2 3 4 5 6 7 |
| **Cupping** | 1 2 3 4 5 6 7 | 1 2 3 4 5 6 7 |

※Reference: 『Lumbar Herniated Intervertebral Disc in Adults: Korean Medicine Clinical Practice Guideline (Korea Institute of Oriental Medicine)』

**2. [Acupuncture, Pharmacopuncture] Rank the following acupoint selection rationales in the order of most frequent use when treating lumbar intervertebral disc displacement patients with acupuncture and pharmacopuncture. (Mark as A~I)**

| **Lumbar intervertebral disc displacement** |
| --- |
| A. Effective acupoints as observed through clinical experience  B. Knowledge acquired through formal education  C. Academic knowledge derived from research articles, clinical practice guidelines  D. Ah-shi points (site of pain)  E. Anatomical structure likely to cause symptoms (e.g. shortened quadratus lumborum, shortened psoas muscles)  F. Spinal levels of pathology as confirmed through imaging (e.g. site of disc herniation)  G. Tender points, trigger points, and other points that elicit a painful response upon palpation  H. Acupoints based on Korean Medicine principles (e.g. GB30, BL40, BL57)  I. Other: __________________________ |
| **1st ( ) 2nd ( ) 3rd ( )** |

※Reference: Revised STandards for Reporting Interventions in Clinical Trials of Acupuncture (STRICTA): Extending the CONSORT Statement

**3. [Acupuncture] The following questions regard acupuncture treatment of lumbar intervertebral disc displacement. Please fill in the blanks for treatments you perform per patient per session. For items on de qi sensation and muscle twitch response, mark the importance of eliciting such response during acupuncture treatment.**

**(Importance: 1=not important at all, 2=unimportant, 3=somewhat unimportant,**

**4=not important, not unimportant, 5=somewhat important, 6=important, 7=very important)**

|  | **Lumbar intervertebral disc displacement** |
| --- | --- |
| **Names of points used [multiple responses allowed]**  **(e.g. GB30, BL40, Huatuo Jiaji points, BL25, GB34, BL23, Ashi points)*** |  |
| **Number of needle insertions** | Average ( ) needles |
| **Depth of needle insertion** | Average ( )cm |
| **Needle retention time** | Average ( ) minutes |
| **Diameter of needle** | Average 0. ( )mm |
| **Needle stimulation [multiple responses allowed]**  **(e.g. Lifting and thrusting (提揷), Holding and twisting (捻轉), Motion Style Acupuncture Treatment)** |  |
| **Percentage of patients treated with electroacupuncture** | About ( )% |
| **How important do you think de-qi sensation is in acupuncture treatment?** | 1 2 3 4 5 6 7 |
| **How important do you think muscle twitch responses are in acupuncture treatment?** | 1 2 3 4 5 6 7 |

*Reference: 『Lumbar Herniated Intervertebral Disc in Adults: Korean Medicine Clinical Practice Guideline (Korea Institute of Oriental Medicine)』

| **Lumbar intervertebral disc displacement** |
| --- |
| A. Ah-shi points  B. Acupoints relevant to symptoms (acupoints related to specific disorder/syndromes)  C. Mu-ja Acupuncture (acupuncture on contralateral side)  D. Five Element Acupuncture  E. Constitution Acupuncture  F. Burning Acupuncture  G. Sa-am Acupuncture  H. Dong-Si Acupuncture  I. Motion Style Acupuncture Treatment (MSAT)  J. Other: __________________________ |
| **1st ( ) 2nd ( ) 3rd ( )** |

**4. [Acupuncture] Rank the following styles of acupuncture in the order you consider most effective for treatment of lumbar intervertebral disc displacement. (Mark as A~J)**

※Reference: 『Acupuncture and Moxibustion (Korean Acupuncture and Moxibustion Medicine Society)』

**5. [Pharmacopuncture] The following questions concern pharmacopuncture treatment for lumbar intervertebral disc displacement. Please fill in the blanks for treatments you perform per patient per session. (Exclude bee venom pharmacopuncture when responding to the following questions)**

|  | **Lumbar intervertebral disc displacement** |
| --- | --- |
| **Most commonly used types of pharmacopuncture**  (e.g. Shinbaro (1, 2, 3), Anti-inflammation Pharmacopuncture, Joongseongouhyul Pharmacopuncture, Scolopendra Pharmacopuncture, Scorpion Pharmacopuncture)* | **1st ( )**  **2nd ( )**  **3rd ( )** |
| **Names of points used [multiple responses allowed]**  (e.g. GB30, BL40, Hyeopcheok (Huatuo Jiaji, EXB2) points, BL25, GB34, BL23, Ah-shi points)* |  |
| **Length of needle** | ( ) cm ~ ( ) cm |
| **Number of acupoint injections per session** | ( ) points ~ ( ) points |
| **Amount of pharmacopuncture solution injected per session** | ( ) cc ~ ( ) cc |
| **Duration of treatment sessions** | ( ) minutes ~  ( ) minutes |
| **Frequency of treatment sessions** | ( ) sessions/week |

*****Reference: 『Lumbar Herniated Intervertebral Disc in Adults: Korean Medicine Clinical Practice Guideline (Korea Institute of Oriental Medicine)』

**6. [Pharmacopuncture] Assuming pharmacopuncture has the 4 following mechanisms of action, rank the factors from most important (influential) in achieving favorable outcomes. (Mark as A~D)**

| A. Acupuncture effects of pharmacopuncture needle (i.e. effect from pharmacopuncture needle itself)  B. Physical stimulation of solution (i.e. irrigation of inflamed area, desensitization effect triggered by pain elicited by injection)  C. Chemical efficacy of solution (i.e. pharmaceutical effect from major ingredients)  D. Placebo effect (i.e. effect from patient anticipation) |
| --- |
| **1st ( ) 2nd ( ) 3rd ( )** |

**7. [Herbal medicine] The following questions concern herbal medicine treatment of lumbar intervertebral disc displacement. Rank the following herbal prescriptions in order of most effective. (Mark as A~K)**

| **Lumbar intervertebral disc displacement** |
| --- |
| A. Chungpa-jun  B. Bojoongikgi-tang (補中益氣湯)  C. Sipjo-tang (十棗湯)  D. Dokhwalgisaeng-tang (獨活寄生湯)  E. Yookmijihwang-tang (六味地黃湯)  F. Shinqi-wan (腎氣丸)  G. Ojeok-san (五積散)  H. Danggwisoo-san (當歸鬚散)  I. Jakyagkamcho-tang (芍藥甘草湯)  J. Hwalhyeoljitong-tang (活血止痛湯)  K. Other: __________________________ |
| **1st ( ) 2nd ( ) 3rd ( )** |

)

※Reference: 『Lumbar Herniated Intervertebral Disc in Adults: Korean Medicine Clinical Practice Guideline (Korea Institute of Oriental Medicine)』 and relevant academic papers

| **Lumbar intervertebral disc displacement** |
| --- |
| **Lumbar spine**  A. Prone lumbosacral joint distraction method  B. Sidelying lumbar ‘pitch and roll’ distraction method  C. Sidelying lumbar extension displacement correction technique  D. Sidelying lumbar flexion displacement correction technique  E. Sidelying lumbar neutral dysfunction correction technique  F. Sitting, lumbar bilateral flexion displacement muscle release/reinforcement technique  G. Spine flexion distraction method: Flexion shift technique  H. Spine flexion distraction method: Sidelying technique  I. Spine flexion distraction method: Circumduction method  J. Spine flexion distraction method: Magnum circulation technique  K. Spine flexion distraction method: Extension technique |
| **Ilium**  L. Prone leg raise ilium correction technique  M. Prone anteriorly rotated ilium correction technique  N. Prone pisiform, metacarpophalangeal joint of 2nd finger inflare-outflare correction technique  O. Prone posteriorly rotated ilium/sidebent sacrum correction technique  P. Sidelying ilium correction technique |
| **Sacrum**  Q. Prone sacrum flexion displacement correction technique  R. Prone sacrum extension displacement correction technique  S. Prone sacrum sidebent rotation displacement correction technique  T. Sidelying sacrum correction technique |
| **Pubis**  U. Supine pubis distraction method  V. Supine upward pubis correction technique  W. Supine downward pubis correction technique |
| **Coccyx**  X. Prone coccyx flexion displacement distraction method |
| **Other**  Y. ( ) |
| **1st ( ) 2nd ( ) 3rd ( )** |

**8. [Chuna] The following Chuna manipulation techniques are relevant to lumbar intervertebral disc displacement. Rank the techniques from most commonly used in your practice. (Mark as A~Y)**

※Reference: 『Chuna Medicine, 2^nd^ edition (Korean Society of Chuna Manual Medicine for Spine & Nerves)』

**[PART 6. Safety]**

**1. Rank how safe you consider the following interventions to be.**

**(1=very unsafe 2=unsafe, 3=somewhat unsafe,**

**4=not safe, but not unsafe, 5=somewhat safe, 6=safe, 7=very safe)**

| **Type of intervention** | **Safety** |
| --- | --- |
| **Acupuncture** | 1 2 3 4 5 6 7 |
| **Pharmacopuncture** | 1 2 3 4 5 6 7 |
| **Bee venom** | 1 2 3 4 5 6 7 |
| **Chuna** | 1 2 3 4 5 6 7 |
| **Herbal medicine** | 1 2 3 4 5 6 7 |
| **Cupping** | 1 2 3 4 5 6 7 |
| **Moxibustion** | 1 2 3 4 5 6 7 |

**2. Rank the following interventions in order of most likely cause of adverse effect(s), and select potential adverse event(s) for each type of treatment. [multiple responses allowed]**

**(e.g. 1st Type of intervention ( C ) – Adverse effect ( a, c, d, e, o ))**

| **Type of intervention** | **Adverse events** |
| --- | --- |
| A. Acupuncture  B. Pharmacopuncture  C. Bee venom  D. Chuna  E. Herbal medicine  F. Cupping  G. Moxibustion | a. Headache  b. Abdominal pain  c. Allergic reactions including pruritus and rashes  d. Anaphylaxis  e. Aggravation of pre-existing pain  f. Gastrointestinal disorders  g. Urination/defecation dysfunction  h. Infection  i. Bleeding and vascular injury  j. Nerve injury  k. Amyotrophy  l. Muscle/tendon/ligament tear  m. Organ injury (e.g. needle penetrating kidney, intestines)  n. Altered consciousness accompanied by vital sign change  o. Pneumothorax  p. Other adverse events: __________________________ |
| **1st Type of intervention ( ) – Adverse event ( )**  **2nd Type of intervention ( ) – Adverse event ( )**  **3rd Type of intervention ( ) – Adverse event ( )** | |

**[PART 7. Clinical decisions]**

**1. The following statements concerning lumbar intervertebral disc displacement can be greatly influenced by patient or physician personal preferences. There are correct responses as supported by current literature. Please provide your opinion on the following statements.**

| **Lumbar intervertebral disc displacement** | |
| --- | --- |
| **Statements** | **Answer choices** |
| 1. For most patients with lumbar intervertebral disc displacement, how likely is doing normal activities to make their herniated disc symptoms worse? | ① Likely  ② Not very likely |
| 2. Without surgery, over time, do back and leg pain caused by lumbar intervertebral disc displacement usually improve, stay the same, or deteriorate? | ① Improves  ② Stays the same  ③ Deteriorates |
| 3. With surgery, over time, do back and leg pain caused by lumbar intervertebral disc displacement usually improve, stay the same, or deteriorate? | ① Improves  ② Stays the same  ③ Deteriorates |
| 4. Can lots of bed rest help relieve pain in some patients with pain caused by lumbar intervertebral disc displacement? | ① Yes  ② No |
| 5. Can over-the-counter pain medicine help relieve pain in some patients with pain caused by lumbar intervertebral disc displacement? | ① Yes  ② No |
| 6. Which treatment is more likely to provide swifter relief from pain caused by lumbar intervertebral disc displacement? | ① Non-invasive care  ② Surgery  ③ Both are similar |
| 7. Of 100 patients who receive surgery for lumbar intervertebral disc displacement, about how many patients will experience equal or more back or leg pain after surgery? | ______ people |
| 8. Of 100 patients who receive surgery for lumbar intervertebral disc displacement, about how many patients will experience serious complications within 3 months of surgery? | ______ people |
| 9. Without surgery, about how many patients with lumbar intervertebral disc displacement will develop permanent loss of motor function severe enough to keep them from walking? | ______ people |
| 10. In the long term (5 years), which treatment is better at relieving pain caused by lumbar intervertebral disc displacement? | ① Non-invasive care  ② Surgery  ③ Both are similar |

※Reference: 『SPINE Volume 37, Number 18**;** Psychometric Evaluation of a Decision Quality Instrument for Treatment of Lumbar Herniated Disc』
